# Mugwort sensitization and asthma as predictors of food-induced anaphylactic shock in children: a retrospective study in Northern China

**DOI:** 10.3389/falgy.2025.1658895

**Published:** 2025-08-21

**Authors:** Zibei Zhou, Juan Zhang, Nini Dai, Zailing Li

**Affiliations:** Department of Pediatrics, Peking University Third Hospital, Beijing, China

**Keywords:** food allergy, anaphylactic shock, children, mugwort, asthma, risk prediction

## Abstract

**Introduction:**

Food-induced anaphylactic shock is the most severe and life-threatening manifestation of food allergy in children. However, its underlying risk factors remain poorly defined, especially in pediatric populations. This study aimed to identify independent predictors of food-induced anaphylactic shock by analyzing clinical and immunological data from a tertiary hospital in Northern China.

**Methods:**

This retrospective study included 68 children (aged 0–16 years) hospitalized for food-induced anaphylaxis at Peking University Third Hospital from May 2018 to May 2025. Demographic data, clinical manifestations, serum-specific immunoglobulin E (sIgE) levels, and comorbidities were collected. Univariate and multivariate logistic regression analyses were performed to identify independent predictors of anaphylactic shock.

**Results:**

Of 68 patients with food-induced anaphylaxis, 14 (20.6%) experienced anaphylactic shock, with 78.6% of these cases occurring in children older than six years. Wheat and fruits were the most common triggers in the shock group (each 28.6%). Subgroup analysis revealed that 88.9% (8/9) of plant-food-triggered shock cases specifically clustered from April to October, aligning with mugwort pollen season. sIgE levels to inhalant allergens, especially mugwort, were significantly higher in the shock group (*P* < 0.05). In multivariate analysis, mugwort sensitization (sIgE levels exceeding 2.83 kU/L, OR = 9.91, 95% CI: 1.27–77.31, *P* = 0.029) and comorbid asthma (OR = 8.11, 95% CI: 1.29–50.98, *P* = 0.026) were identified as independent predictors of anaphylactic shock.

**Conclusions:**

Mugwort sensitization and asthma are independent risk factors for food-induced anaphylactic shock in children. Early identification of these risk markers may support early risk stratification and seasonal dietary interventions.

## 1 Introduction

Food allergy (FA) is an adverse immune response to specific food antigens, and its global prevalence has risen substantially in recent decades, posing a significant public health challenge (1–4). When the respiratory or circulatory systems are involved, FA reactions can rapidly escalate to distributive shock, leading to multiorgan failure or death. Despite its severity, food-induced anaphylactic shock remains unpredictable in pediatric populations.

Previous studies have suggested that various factors may influence the severity of allergic reactions, including the type and number of food allergens (1, 5, 6), age (7), underlying atopic diseases [such as asthma (8, 9)], and cofactors such as exercise (10) or the use of nonsteroidal anti-inflammatory drugs (NSAIDs) (11). Moreover, up to 60% of FA reactions may be associated with cross-reactivity to inhalant allergens (12). In Northern China, plant-derived foods—especially wheat and fruits—are common food allergens (13) and are significantly linked to pollinosis (14). The prevalence of wheat allergy among children in Northern China is 3.6% (15), while the self-reported prevalence of fruit allergies in children aged 0–5 years is 1.55% (16). Aeroallergens like mugwort pollen are highly prevalent and may contribute to enhanced allergic responses due to molecular cross-reactivity with plant-derived foods (17). However, most current data are derived from adult populations, and there is a lack of pediatric-specific evidence, particularly regarding risk factors for the development of anaphylactic shock in children. This knowledge gap limits our ability to identify high-risk children and implement early intervention strategies.

In this retrospective study, we analyzed clinical and laboratory data from pediatric patients with food-induced anaphylaxis at a tertiary hospital in Northern China. Using univariate and multivariate logistic regression, we aimed to identify independent risk factors for food-induced anaphylactic shock in children, when compared to non-shock anaphylaxis cases. Our goal is to inform clinical risk stratification, guide preventive strategies, and identify high-risk children who may benefit from targeted education or desensitization therapy.

## 2 Materials and methods

### 2.1 Study design and participants

This retrospective study enrolled pediatric patients (aged 0–16 years) diagnosed with food-induced anaphylaxis and hospitalized at the Pediatric Department of Peking University Third Hospital from May 2018 to May 2025. The study protocol was approved by the hospital's Ethics Committee (Approval No. M20250454) and conducted in accordance with the Declaration of Helsinki.

### 2.2 Data collection

Patient data were retrieved from the hospital's electronic medical record system (Jiahe, Beijing, China). Variables collected included demographic characteristics (age, sex), month of symptom onset, latency between food exposure and symptom onset, clinical manifestations, history of allergic diseases, family history of allergies (first-degree relatives), and laboratory test results (total serum IgE, allergen-specific IgE, and skin prick test results).

### 2.3 Diagnostic criteria

Anaphylaxis was diagnosed based on the 2020 World Allergy Organization (WAO) criteria (18). Food-induced anaphylactic shock was defined as anaphylaxis accompanied by hypotension, corresponding to Grade 5 in the WAO classification. Hypotension was defined using the 2021 European Academy of Allergy and Clinical Immunology guidelines (19): systolic blood pressure below the age-specific threshold or a >30% decrease from baseline (e.g., <70 mmHg in infants aged 1 month–1 year, <70 + 2× age in years mmHg for children aged 1–10 years, <90 mmHg for adolescents aged 11–17 years). Food triggers were identified based on patient history and confirmed via skin prick testing and/or serum sIgE detection. In cases where NSAIDs were suspected as cofactors, medication history was reviewed, and oral provocation tests under fasting conditions were conducted to exclude NSAID hypersensitivity. All diagnoses were confirmed by board-certified allergy and immunology specialists.

### 2.4 Statistical analysis

Statistical analyses were performed using SPSS version 27.0. Continuous variables with non-normal distribution were expressed as median (interquartile range) [M (P25, P75)] and compared using the Mann–Whitney *U* test. Categorical variables were presented as counts (percentages) and compared using the chi-square test or Fisher's exact test as appropriate. A *P*-value <0.05 was considered statistically significant. To identify potential risk factors for food-induced anaphylactic shock in children, univariate analysis was first performed using the chi-square test. Receiver operating characteristic curves were generated for continuous variables to determine optimal cut-off values based on the Youden index, and variables were then dichotomized by these thresholds for further chi-square testing. Variables with *P* < 0.05 in univariate analysis were entered into a multivariate logistic regression model to identify independent risk factors, with significance set at *P* < 0.05.

## 3 Results

### 3.1 Patient characteristics

**Figure 1:**
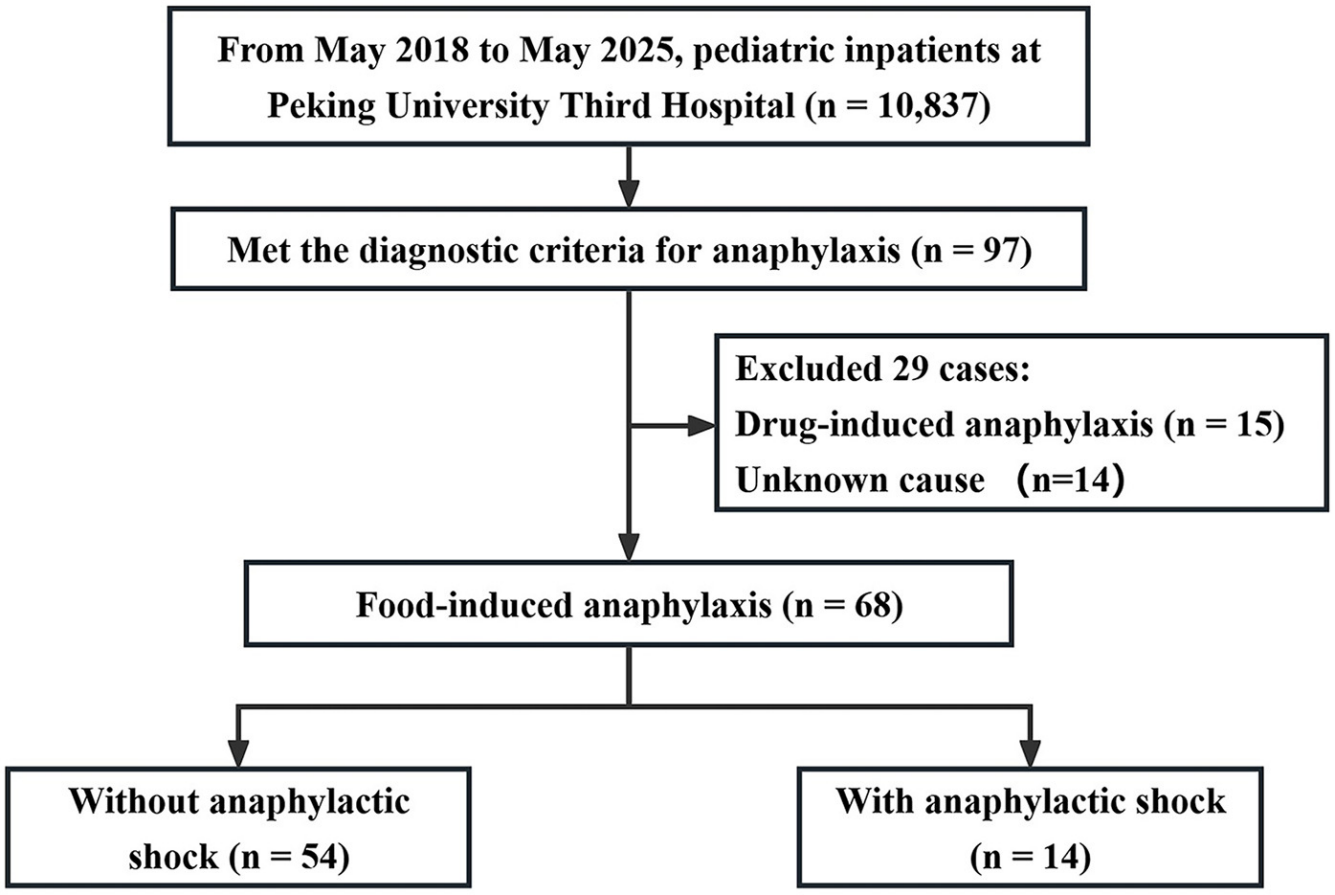
Flowchart of patient inclusion. Study enrollment diagram showing the screening and inclusion of 68 pediatric patients with food-induced anaphylaxis, including 14 cases with anaphylactic shock and 54 without shock.

Among 10,837 pediatric admissions during the study period, 97 were diagnosed with anaphylaxis. Of these, 68 cases (70.1%) were attributed to food-induced anaphylaxis and included in the final analysis. Within this group, 14 patients (20.6%) developed anaphylactic shock, while 54 (79.4%) did not. A majority of patients were male (63.2%). Figure 1 summarizes the study enrollment process.

### 3.2 Age distribution

**Figure 2:**
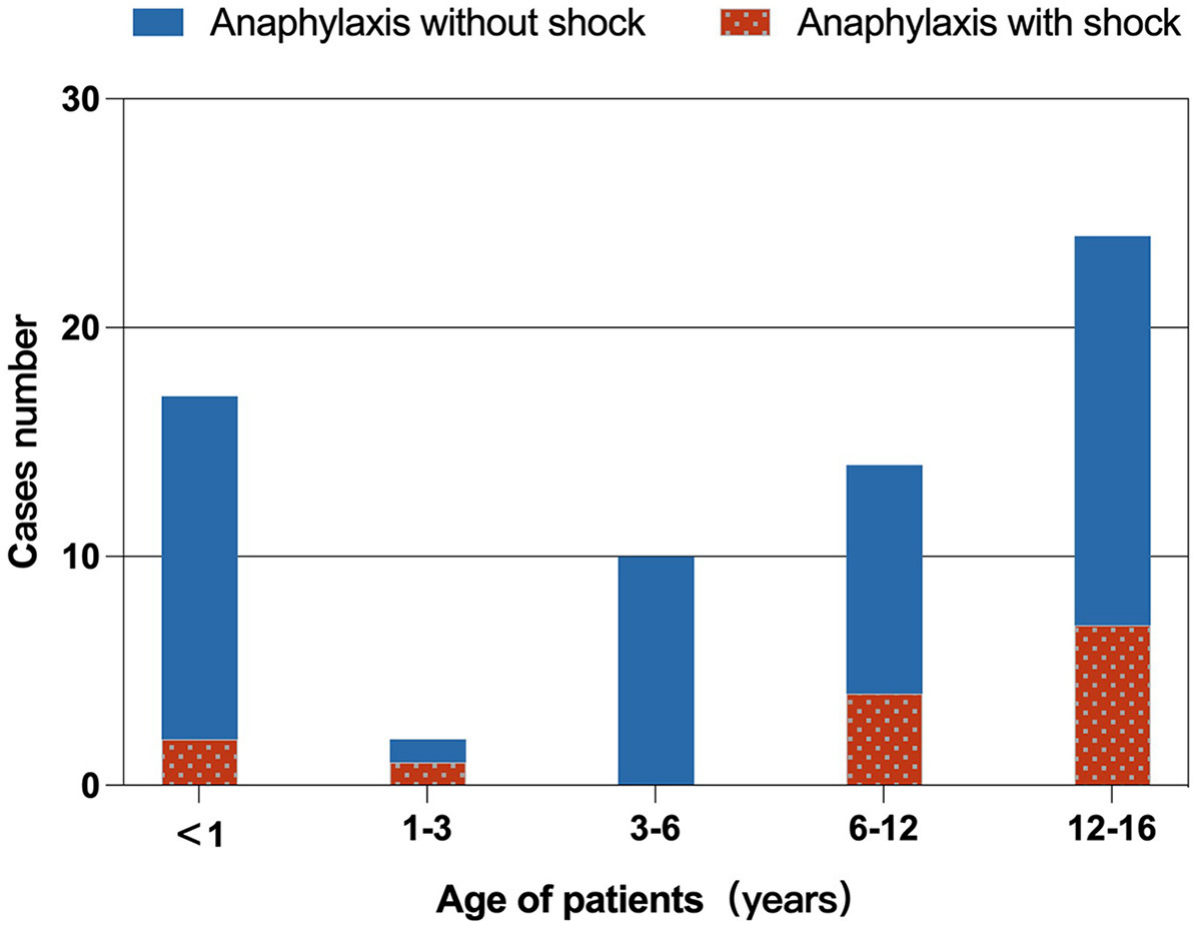
Age distribution of children with food-induced anaphylaxis. Anaphylactic shock occurred more frequently in children over 6 years of age (11/14, 78.6%).

The age distribution of children with food-induced anaphylaxis is shown in Figure 2. The non-shock group ranged from 1 month and 17 days to 15 years, while the shock group ranged from 4 months and 18 days to 15 years. Notably, most cases of shock occurred in children older than six years (11/14, 78.57%).

### 3.3 Food triggers

Table 1 presents the distribution of food triggers. Fruits (22.1%) and dairy products (19.1%) were the most common allergens overall. In the shock group, wheat and fruits were the predominant triggers (each 28.6%), whereas dairy products (12/54, 22.22%) and fruits (11/54, 20.37%) were most frequent in the non-shock group. No statistically significant differences in food allergen distribution were observed between the two groups (*P* > 0.05).

**Table 1:** Distribution of food allergen triggers in shock and non-shock groups.

Food Allergen	Total (*n* = 68)	Non-shock (*n* = 54)	Shock (*n* = 14)	*P*
Wheat [*n* (%)]	11 (16. 18%)	7 (12.96%)	4 (28.57%)	0.219
Milk or dairy products [*n* (%)]	13 (19.12%)	12 (22.22%)	1 (7.14%)	0.275
Egg [*n* (%)]	3 (4.41%)	3 (5.56%)	0 (0.00)	1.000
Fruits [*n* (%)]	15 (22.06%)	11 (20.37%)	4 (28.57%)	0.491
Peach [*n* (%)]	3 (4.41%)	2 (3.7%)	1 (7.14%)	0.505
Cornus fruit [*n* (%)]	4 (5.88%)	4 (7.41%)	0 (0.00)	0.574
Mango [*n* (%)]	1 (1.47%)	0 (0.00)	1 (7.14%)	0.206
Lychee [*n* (%)]	2 (2.94%)	2 (3.7%)	0 (0.00)	1.000
Dragon fruit [*n* (%)]	2 (2.94%)	2 (3.7%)	0 (0.00)	1.000
Coconut [*n* (%)]	1 (1.47%)	0 (0.00)	1 (7.14%)	0.206
Melon [*n* (%)]	1 (1.47%)	0 (0.00)	1 (7.14%)	0.206
Apple [*n* (%)]	1 (1.47%)	1 (1.85%)	0 (0.00)	1.000
Nuts [*n* (%)]	10 (14.71%)	9 (16.67%)	1 (7.14%)	0.674
Walnut [*n* (%)]	4 (5.88%)	3 (5.56%)	1 (7.14%)	1.000
Sesame [*n* (%)]	4 (5.88%)	4 (7.41%)	0 (0.00)	0.574
Peanut [*n* (%)]	1 (1.47%)	1 (1.85%)	0 (0.00)	1.000
Hazelnut [*n* (%)]	1 (1.47%)	1 (1.85%)	0 (0.00)	1.000
Seafood [*n* (%)]	5 (7.35%)	4 (7.41%)	1 (7.14%)	1.000
Shrimp [*n* (%)]	4 (5.88%)	4 (7.41%)	0 (0.00)	0.574
Codfish [*n* (%)]	1 (1.47%)	0 (0.00)	1 (7.14%)	0.206
Other foods [*n* (%)]	2 (2.94%)	2 (3.7%)	0 (0.00)	1.000
Green beans [*n* (%)]	1 (1.47%)	1 (1.85%)	0 (0.00)	1.000
Beef [*n* (%)]	1 (1.47%)	1 (1.85%)	0 (0.00)	1.000
Mixed foods [*n* (%)]	9 (13.24%)	6 (11.11%)	3 (21.43%)	0.377

Note: Fisher's exact test was used for all comparisons.

### 3.4 Seasonal patterns and aeroallergen sensitization

**Figure 3:**
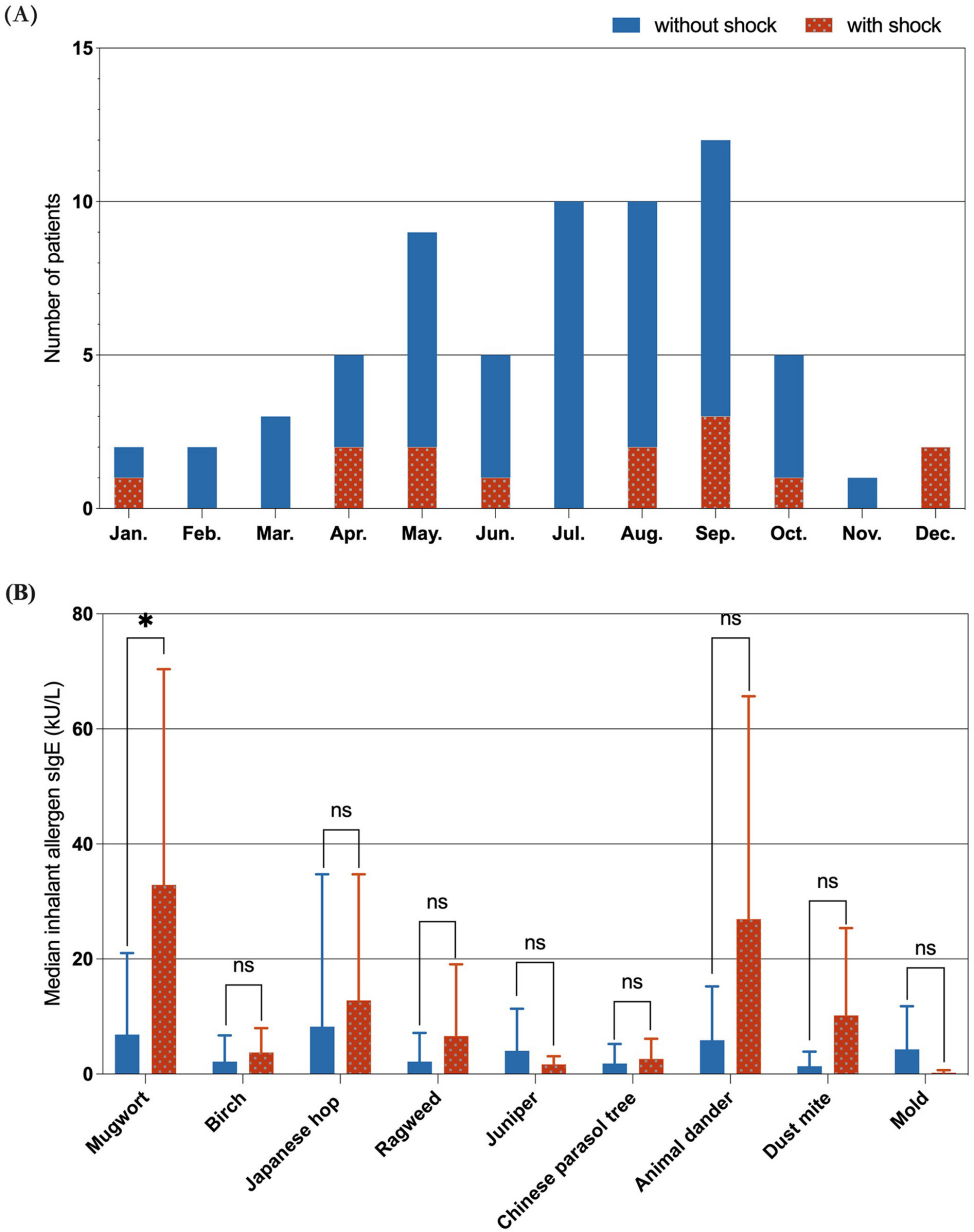
Seasonal distribution and inhalant allergen sensitization. **(A)** Cases were concentrated between April and October, with a peak in September. **(B)** Mugwort sensitization was significantly more common in the shock group (*P* < 0.05), other inhalant allergens showed no significant difference. (In the figure, “ns” represents *P* > 0.05; “*” represents *P* < 0.05).

Figure 3A illustrates the monthly distribution of food-induced anaphylaxis cases, with a marked increase from April to October and a peak in September. While shock reactions were more equally distributed throughout the year, subgroup analysis revealed that 88.9% (8/9) of plant-food-triggered shock cases (including wheat, peach, walnut, melon, mango and coconut) specifically clustered from April to October. Figure 3B compares the median levels of sIgE to common inhalant allergens between the two groups. Mugwort sensitization was significantly more common in the shock group (*P* < 0.05), while no significant differences were found for other aeroallergens, including ragweed, birch, Japanese hop, animal dander, dust mites, and molds.

### 3.5 Predictive value of mugwort sIgE: receiver operating characteristic (ROC) analysis

**Figure 4:**
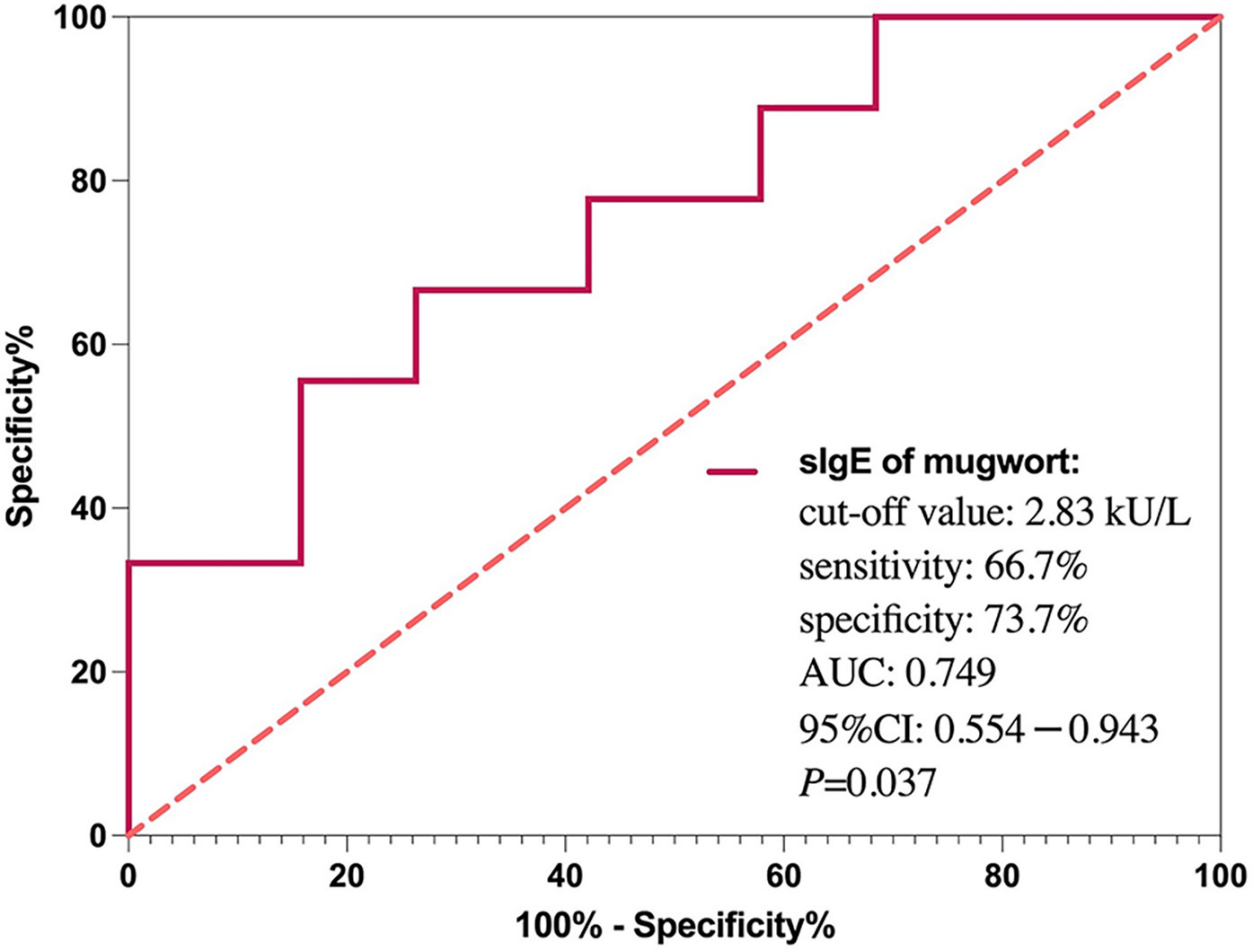
ROC curve for mugwort-specific IgE predicting anaphylactic shock. The area under the curve (AUC) was 0.749. A cut-off value of 2.83 kU/L yielded 66.7% sensitivity and 73.7% specificity.

To assess the predictive performance of mugwort sIgE, ROC curve was generated (Figure 4). The area under the curve (AUC) was 0.749, indicating good discriminative ability. The optimal cut-off value for mugwort sIgE was determined to be 2.83 kU/L, with a sensitivity of 66.7% and specificity of 73.7% for predicting anaphylactic shock.

### 3.6 Univariate analysis of risk factors for food-induced anaphylactic shock

As shown in Table 2, univariate logistic regression identified comorbid asthma [Odds ratio [OR] = 6.80, 95% Confidence interval [CI]: 1.31–35.17, *P* = 0.022] and mugwort sensitization (sIgE levels exceeding 2.83 kU/L, OR = 8.40, 95% CI: 1.26–56.07, *P* = 0.028) as significant risk factors for anaphylactic shock. Other variables, including coexisting other allergic diseases (except asthma), cofactor exposure, age, food type and family history of allergic diseases, were not significantly associated with anaphylactic shock.

**Table 2:** Univariate analysis of risk factors for food-induced anaphylactic shock.

Risk factor	*β*	S. E	*Z*	*P*	OR (95%CI)
Physical exercise (cofactor)	1.16	0.73	1.59	0.113	3.20 (0.76–13.46)
NSAIDs (cofactor)	−16.33	1,615.10	−0.01	0.992	0.00 (0.00∼Inf)
Age >6 years	1.23	0.71	1.74	0.083	3.40 (0.85–13.58)
Time from exposure to symptom >30 min	0.28	0.63	0.44	0.661	1.32 (0.38–4.56)
Plant-based food as the trigger	0.84	0.65	1.29	0.196	2.32 (0.65–8.32)
Family history of allergic diseases	−0.29	0.60	−0.48	0.634	0.75 (0.23–2.45)
Comorbid allergic diseases	0.31	0.84	0.37	0.712	1.36 (0.26–7.08)
Comorbid eczema/atopic dermatitis	0.21	0.60	0.35	0.724	1.24 (0.38–4.05)
Comorbid allergic rhinitis	0.04	0.67	0.06	0.953	1.04 (0.28–3.83)
Comorbid asthma	1.92	0.84	2.29	0.022	6.80 (1.31–35.17)
Comorbid drug allergy	1.47	1.05	1.40	0.163	4.33 (0.55–33.93)
Mugwort sensitization (sIgE > 2.83 kU/L)	2.13	0.97	2.20	0.028	8.40 (1.26–56.07)

### 3.7 Multivariate analysis of risk factors for food-induced anaphylactic shock

In multivariate logistic regression (Table 3), both mugwort sensitization (OR = 9.91, 95% CI: 1.27–77.31, *P* = 0.029) and asthma (OR = 8.11, 95% CI: 1.29–50.98, *P* = 0.026) remained independent predictors of anaphylactic shock.

**Table 3:** Multivariate logistic regression analysis of independent risk factors.

Risk factor	*β*	S. E	*Z*	*P*	OR (95%CI)
Mugwort sensitization (sIgE > 2. 83 kU/L)	2.29	1.05	2.19	0.029	9.91 (1.27–77.31)
Comorbid asthma	2.09	0.94	2.23	0.026	8.11 (1.29–50.98)

## 4 Discussion

In this retrospective analysis of 68 pediatric cases of food-induced anaphylaxis, we identified two independent risk factors for anaphylactic shock in children from Northern China: elevated sensitization to mugwort pollen (sIgE levels exceeding 2.83 kU/L) and a history of asthma, when compared to non-shock anaphylaxis cases. In contrast, food allergen type, cofactor exposure (e.g., physical exertion or NSAIDs use), and other comorbid allergic diseases (except asthma) did not show significant associations with shock.

Regional variations in environmental exposures, dietary habits, and climate across China contribute to distinct allergic sensitization patterns (12). In Northern China, mugwort pollen is the predominant aeroallergen during late summer and early autumn (20). With over 180 Artemisia species documented (21), cross-reactive molecules such as non-specific lipid transfer proteins (nsLTPs) and profilins—shared between mugwort pollen and many plant-derived foods—can trigger allergic responses. These proteins are highly stable, resisting both digestion and heat, and are recognized as potent allergens (17, 22). In our study, plant-food-triggered shock cases specifically clustered from April to October, aligning with mugwort pollen season, suggesting a potential “priming–amplification” effect of seasonal inhalant sensitization on FA severity. Among the aeroallergens evaluated, only mugwort sensitization was independently associated with anaphylactic shock (*P* < 0.05). A mugwort sIgE level exceeding 2.83 kU/L was associated with nearly tenfold increased risk of shock (OR = 9.91), highlighting the clinical importance of mugwort screening. These findings underscore the need for targeted counseling and avoidance strategies in mugwort-sensitized children, especially regarding consumption of cross-reactive foods during high-pollen periods.

Asthma history also emerged as an independent predictor of anaphylactic shock. Many asthmatic patients in our cohort had poorly controlled disease and suboptimal adherence to inhaled corticosteroid therapy. FA and asthma may share common sensitization pathways and inflammatory mechanisms, with potential synergistic effects. Shared type 2 immune pathways and epithelial barrier dysfunction may contribute to enhanced allergen absorption and anaphylaxis (23). Additionally, elevated Th2 cytokines such as IL-4 and IL-13 in poorly controlled asthma may upregulate IgE synthesis and mast cell activation (24), further compounding the risk of anaphylactic shock. These insights support a dual-management approach in pediatric patients with asthma: on one hand, achieving optimal asthma control, such as improving adherence through single maintenance and reliever therapy to enhance asthma management; on the other hand, assessing and mitigating the risk of food-induced allergic reactions.

Although cofactors such as physical activity and NSAID use are recognized exacerbators of anaphylaxis in adults (25–27), our data did not show a significant association between these factors and anaphylactic shock in children. This aligns with findings from Casas-Saucedo et al. (28) and may reflect the lower intensity of physical exertion and limited NSAID use in pediatric populations. Furthermore, intrinsic host factors—such as genetic susceptibility (29) and reduced compensatory mechanisms (30, 31) —may play a more prominent role in pediatric anaphylaxis severity than external triggers.

Our results emphasize the importance of incorporating regional inhalant allergen profiles into pediatric allergy risk assessments. In areas like Northern China, where mugwort pollen exposure is common, measuring sIgE levels and evaluating cross-reactivity with food allergens should be part of routine care. Children with coexisting asthma and mugwort sensitization represent a particularly high-risk group and should be prioritized for early intervention, including seasonal dietary guidance and personalized management plans. Unlike Western cohorts, where peanut, tree nuts, or milk are primary triggers (32), our findings point to plant-based foods—particularly fruits and wheat—as the leading causes of food-induced anaphylactic shock in Northern Chinese children. This regional difference underscores the importance of context-specific prevention strategies.

Several limitations warrant consideration. First, the retrospective, single-center design and relatively small number of shock cases (*n* = 14) may limit statistical power and generalizability. Second, due to data limitations, we could not assess the influence of food type, quantity, or component-resolved allergen profiles. Future multicenter studies with larger sample sizes are needed to further explore the interaction between food allergens and environmental factors. Moreover, the integration of component-resolved diagnostics may enhance risk stratification by identifying specific sensitizing molecules and may facilitate the development of targeted immunotherapy or biologic interventions for high-risk populations.

## 5 Conclusion

This study identifies mugwort pollen sensitization and a history of asthma as independent risk factors for food-induced anaphylactic shock in children from Northern China. These findings underscore the need to incorporate regional aeroallergen profiles and comorbid respiratory conditions into pediatric allergy risk assessments. Routine screening for mugwort sensitization—particularly during peak pollen seasons—and strict asthma control may reduce the risk of life-threatening anaphylactic events. Region-specific strategies, including seasonal dietary counseling and personalized allergy management, are essential for high-risk populations. Future multicenter and prospective studies integrating molecular diagnostics are warranted to further refine risk prediction models and improve early intervention in pediatric food allergy.

## Data Availability

The raw data supporting the conclusions of this article will be made available by the authors, without undue reservation.
